# The Role of Artificial Intelligence in Optimizing Diagnosis in Prostate Cancer—A Narrative Review

**DOI:** 10.3390/jcm15187189

**Published:** 2026-09-16

**Authors:** Razvan George Rahota, Andrei Vlad Badulescu, Bogdan Adrian Buhas, Margareta Moga, Diana Vaidean, Alina Popa, Guillaume Ploussard

**Affiliations:** 1 Department of Urology, Medicover Pelican Hospital, Faculty of Medicine and Pharmacy, University of Oradea, 410087 Oradea, Romania; drrazvanrahota@gmail.com; 2 Doctoral School of Biomedical Sciences, Faculty of Medicine and Pharmacy, University of Oradea, 410087 Oradea, Romania; 3 Department of Urology, Groupe Hospitalier Diaconesses Croix Saint-Simon, 75020 Paris, France; 4 University of Medicine and Pharmacy “Iuliu Hatieganu”, 400347 Cluj-Napoca, Romania; 5 Clinical Hospital of Rehabilitation Baile Felix, Faculty of Medicine and Pharmacy, University of Oradea, 410087 Oradea, Romania; 6 Clinical Emergency County Hospital, Faculty of Medicine and Pharmacy, University of Oradea, 410087 Oradea, Romania; 7 Service d ‘Urologie, Oncopole Claudius Regaud, 31059 Toulouse, France; g.ploussard@gmail.com; 8 UROSUD, Clinique La Croix du Sud, 31130 Toulouse, France

**Keywords:** artificial intelligence, prostate cancer, radiomics, pathomics, machine learning, deep learning, multiparametric magnetic resonance imaging, PSMA PET, digital pathology

## Abstract

Artificial intelligence (AI) is increasingly being investigated in prostate cancer (PCa) diagnosis and characterization, offering novel approaches to improve detection and risk stratification. This narrative review summarizes current evidence regarding the application of AI across the major stages of PCa management, with particular emphasis on radiomics and pathomics. Radiomics enables the extraction of high-dimensional quantitative features from medical imaging modalities, including ultrasound, computed tomography, multiparametric magnetic resonance imaging (mpMRI), and prostate-specific membrane antigen positron emission tomography (PSMA PET), providing imaging biomarkers that extend beyond conventional visual interpretation. Numerous studies have demonstrated that AI-based radiomic models improve the detection of clinically significant PCa, characterize tumor aggressiveness, predict extracapsular extension, and support individualized treatment selection. Among available imaging modalities, mpMRI remains the cornerstone for radiomics owing to its superior soft-tissue characterization, whereas PSMA PET radiomics has shown particular promise for assessing biologically aggressive disease and metastatic spread. Pathomics has further expanded the role of AI by enabling automated tumor detection, grading, quantification, and identification of adverse pathological features, with promising performance reported in selected retrospective validation studies. Despite encouraging results, widespread clinical implementation remains limited by heterogeneous imaging protocols, variability in data acquisition and annotation, lack of standardized workflows, insufficient prospective multicenter validation, and ethical and regulatory challenges. The aim of this review was to summarize current evidence on AI-based imaging analysis, radiomics, and pathomics for PCa detection, characterization, risk stratification, and pathological assessment, while highlighting the methodological challenges that currently limit clinical implementation.

## 1. Introduction

**Figure 1:**
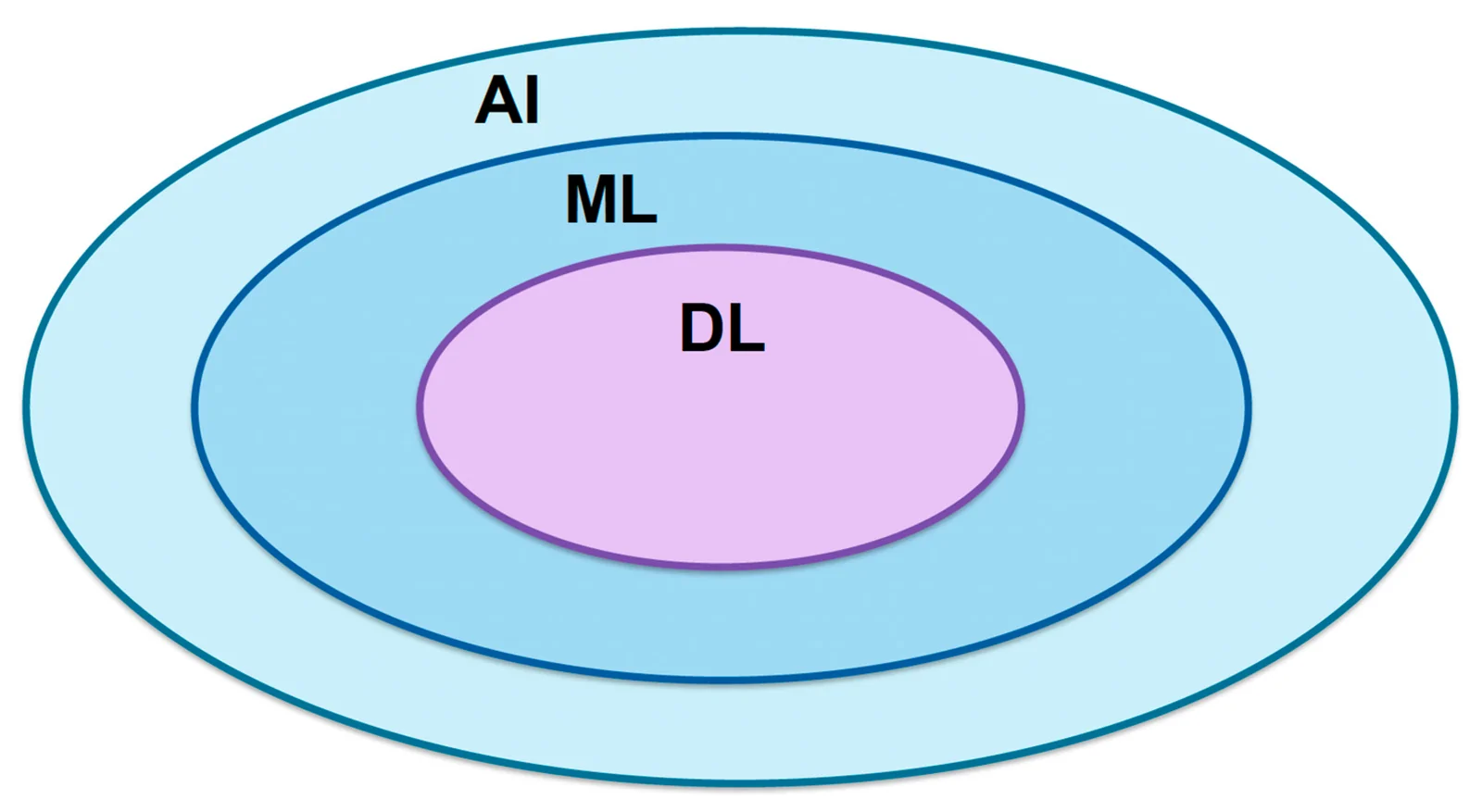
Conceptual relationship between AI, ML, and DL.

Artificial intelligence (AI) is defined by replicating human intelligence in machines that are then programmed to learn, evolve and adapt like humans (Figure 1). It is a vast area that encompasses machine learning (ML), where computers are able to acquire and interpret data (structured and unstructured) or models, and deep learning (DL) in order to perform complex tasks using different neural networks [1]. The most significant ones for medical application are convolutional neural networks that are designed for processing and analyzing images such as histopathology slides, ultrasound or radiological scans, thus having the potential of optimizing patient clinical outcomes [2,3,4]. Through AI assistance, big data characterized by high volume, high velocity and high variety information can be rapidly, effectively and cost-efficiently evaluated, generating clinical predictions [5].

Prostate cancer (PCa) is the most diagnosed malignancy in men worldwide, with an increasing incidence and prevalence in developed countries [6,7], mostly due to the implementation of screening methods (digital rectal examination, PSA testing) and increasing accuracy of the diagnostic tools such as multiparametric prostate MRI (mpMRI) or fusion-targeted prostate biopsies [8,9,10].

Nonetheless, the cancer-specific mortality rate in PCa remains high, even in the era of robotic-assisted surgery, where robot-assisted radical prostatectomy (RARP) has become the gold-standard surgical treatment for localized PCa [11]. This opens the question of whether or how AI can improve diagnostic and treatment pathways for PCa in order to achieve higher detection accuracy of clinical significant PCa and lower mortality rates.

In urology, the benefit of implementing AI for improving diagnosis and treatment of PCa refers to the ability of processing large volumes of patient data such as medical imaging reports and histopathological biopsy data, providing risk assessment and improving personalized patient treatment [12]. Among the applications of AI in PCa, quantitative imaging analysis and computational pathology have received particular attention. Radiomics enables the extraction and analysis of quantitative imaging features, whereas pathomics applies computational methods to digitized histopathological specimens. These approaches may also be integrated with molecular and genomic information to develop multimodal models of tumor phenotype and aggressiveness. The aim of this narrative review is to summarize current evidence on AI-based imaging analysis, radiomics, and pathomics for PCa detection, characterization, risk stratification, and pathological assessment.

This narrative review was based on a literature search conducted using PubMed/MEDLINE, Scopus, and Web of Science. The search focused on studies published up to June 2026 and used combinations of the terms “prostate cancer”, “artificial intelligence”, “machine learning”, “deep learning”, “radiomics”, “pathomics”, “digital pathology”, and “multiparametric MRI”. Reference lists of relevant original articles and reviews were also screened to identify additional studies of interest. Priority was given to peer-reviewed studies evaluating clinically relevant applications of AI in prostate cancer diagnosis, imaging-based characterization, risk stratification, histopathological assessment, and prediction of oncological outcomes. Studies were selected based on their clinical relevance, methodological contribution, validation strategy, and relevance to the main topics of this narrative review. Particular attention was given to studies reporting quantitative diagnostic or prognostic performance and to landmark studies illustrating the development or clinical implementation of AI-based approaches. Given the narrative and non-systematic design of this review, study selection may be subject to selection bias, and the included literature should therefore be considered representative rather than exhaustive.

## 2. The Use of Radiomics in PCa Diagnosis

### 2.1. What Is Radiomics?

Traditionally, the diagnostic pathway of PCa consists of biopsy and histopathological examination of the obtained specimens; however, this method has its limitations [13]. Biopsies can be invasive, make the patient uncomfortable and pose a risk for complications such as bleeding and infection after the procedure, especially in the case of a transrectal approach; these risks can be reduced by using the transperineal approach and/or performing fusion-targeted biopsies with fewer cores being harvested [14]. Moreover, the histopathology report of the tissue samples is reader-dependent and relies greatly on the experience of the pathologist, which can generate potential errors in diagnosis [15]. Even with the widespread adoption of mpMRI for early detection of suspicious lesions in the prostate that are characterized by a PIRADS score and, if necessary, biopsied using fusion techniques, results are still suboptimal in many centers mostly due to reader variability and radiologists’ experience. In order to overcome this pitfall, the information coming from radiological studies can be better assessed using radiomics.

**Figure 2:**
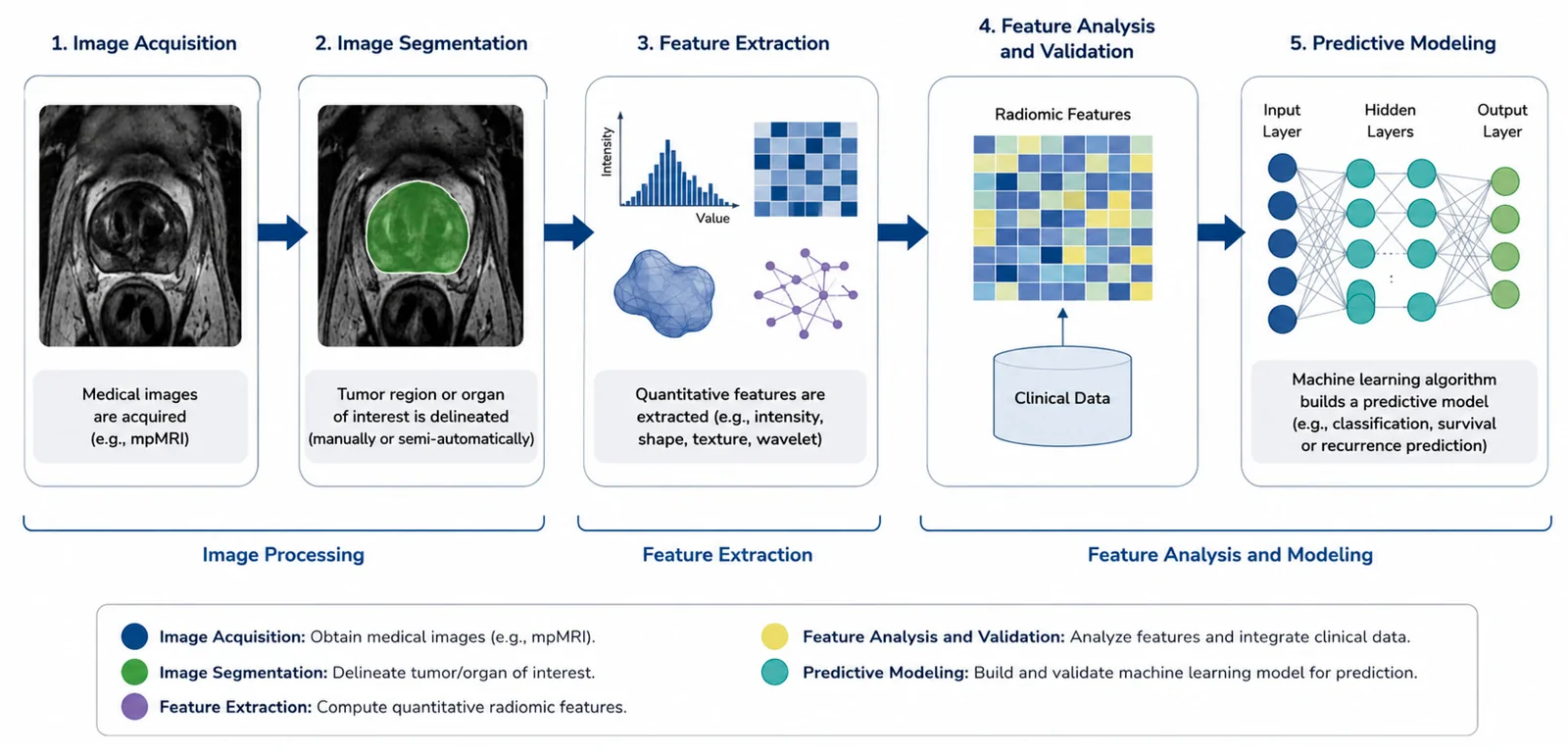
Visual description of the radiomics process, from acquisition of images to predictive modeling.

Radiomics is a quantitative image-analysis methodology that enables the extraction of high-dimensional features from medical images, including CT, MRI, ultrasound, and PET/CT. These features may describe lesion intensity, shape, texture, and spatial heterogeneity and can subsequently be analyzed using conventional statistical approaches or incorporated into machine-learning models for classification, prediction, and correlation with clinical outcomes. Therefore, although radiomics is frequently integrated into AI-based pipelines, radiomics and AI should not be considered synonymous [16]. Radiomics was first described by Gillies et al. and is characterized by a sequential workflow (Figure 2) that implies the acquisition of high-quality images (e.g., from prostate mpMRI), segmentation of these images with the delimitation of the region of interest, extraction of quantitative features where the most important data is encompassed in predictive models, analysis of these features, and correlation with clinical information, which is followed by validation of the results in order to generate prediction outcomes [17,18,19,20].

Lambin et al. [21] developed a score for assessing the quality of radiomics data interpretation referred to as the Radiomics Quality Score (RQS), revised in 2025. It evaluates all the steps of the radiomics process above, aiming to improve high-quality end results and ensure a standardized methodology for researchers and clinicians, similarly to the prostate imaging quality (PI-QUAL) score that radiologists use in prostate mpMRI [22]. Because acquisition of images from radiological studies is the first step in radiomics, having high-quality data and using the correct imaging modality for initial evaluation of prostate cancer is of utmost importance for achieving the best results and predicting outcomes. Multiple imaging studies can be used for radiomics, such as ultrasound (US), computed tomography (CT) scan, MRI, and positron emission tomography scan (PET-CT), but for prostate cancer, mpMRI is considered to be the best radiological exploration to extract tumor information [23].

### 2.2. Imaging Studies in PCa

US remains a first-setting diagnostic evaluation tool for patients with a biochemical suspicion of PCa because it is widely available in almost all clinical practices, non-invasive, and cheap, and can be repeated without irradiation risks, but the detection of PCa is low, around 40% for traditional greyscale US [24]. The detection rate of clinically significant PCa can be improved using advanced ultrasound techniques, including contrast-enhanced ultrasound, Doppler imaging, and elastography [25,26]. More recently, high-resolution micro-ultrasound (micro-US) has emerged as an additional imaging technique for prostate cancer detection. Operating at substantially higher frequencies than conventional transrectal ultrasound, micro-US provides improved spatial resolution and enables visualization of subtle alterations in prostatic architecture. In the multicenter randomized OPTIMUM trial, micro-US-guided biopsy was noninferior to MRI/conventional ultrasound fusion-guided biopsy for the detection of clinically significant PCa, defined as ISUP Grade Group ≥2 disease [27]. These findings support micro-US as an alternative image-guidance strategy for prostate biopsy, although its role in dedicated radiomics and AI-based analysis remains less established. Ou et al. [28] developed a transrectal US-based radiomics model to improve pre-biopsy prostate cancer prediction using grayscale ultrasound images obtained from 196 patients undergoing initial prostate biopsy. Following manual delineation of the lesion, more than 1000 radiomic features were extracted, and least absolute shrinkage and selection operator (LASSO) regression identified five features that constituted a radiomics score. Multivariable logistic regression demonstrated that the radiomics score, together with age, total prostate-specific antigen and prostate volume, independently predicted prostate cancer. An integrated nomogram combining the radiomics score with these clinical variables achieved an AUC of 0.835 in the validation cohort, significantly outperforming the clinical model based solely on conventional parameters (AUC 0.752; *p* = 0.04).

Conventional CT has limited sensitivity for detecting lymph-node metastases in PCa because nodal assessment relies predominantly on morphological criteria, particularly lymph-node size and shape. Commonly used size thresholds are based on short-axis diameter and vary according to anatomical location; consequently, metastatic deposits may be present in morphologically normal-sized lymph nodes, whereas enlarged nodes may be benign. This limitation contributes to the relatively poor sensitivity of conventional CT for nodal staging [29,30,31,32]. Osman et al. [33] evaluated radiomic features extracted from non-contrast radiotherapy-planning CT for Gleason grade and clinical risk stratification. Although high AUC values were obtained during model development, performance was lower on validation, with AUCs of 0.75 for distinguishing low- from high-risk disease and 0.70 for distinguishing Gleason score 7 from >7 disease. Bosetti et al. [34] investigated longitudinal cone-beam CT radiomics in a smaller cohort and reported cross-validated AUCs of 0.83 for risk group classification and approximately 0.80–0.82 for Gleason score classification. In contrast, Peeken et al. [35] addressed a different clinical endpoint, developing a CT-based radiomics model for the detection of lymph-node metastases in patients undergoing PSMA-radioguided surgery. These findings illustrate the potential diversity of CT-based radiomics applications but should be interpreted cautiously because of differences in endpoints, cohort size, and validation strategies.

Prostate mpMRI with the PIRADS classification score has emerged as the best imaging diagnostic tool for PCa, with a sensitivity of 93% for clinically significant PCa, according to the PROMIS study [36]; moreover, accuracy of prostate biopsy can be improved by using MRI-US fusion techniques. In addition, mpMRI provides high-quality images and data for radiomics studies, outperforming the PIRADS classification in terms of sensitivity, specificity and AUC (90% vs. 80%, 70% vs. 47% and 0.85 vs. 0.72 respectively, *p* < 0.05) for the detection of PCa [37]. This is particularly important for unequivocal PIRADS 3 lesions described on mpMRI that can hide clinically significant PCa in up to 40% of cases [38]. Implementation of radiomics in mpMRI of the prostate can help in distinguishing those PIRADS 3 lesions that harbor clinically significant PCa and require active treatment (surgery or radiotherapy) that will impact cancer specific survival. Another advantage of applying radiomics in prostate mpMRI is for patients on an active-surveillance pathway, to reduce the number of unnecessary prostate biopsies. However, a few drawbacks can be identified for these algorithms. Firstly, images and data from mpMRI come from multiple machines, thus making it difficult for an universal radiomics algorithm to be developed [39]. Secondly, large sets of data are required to distinguish between low-/intermediate-/high-risk PCa and also between various ranges of the Gleason score, differences that carry important clinical implications and allow for a personalized treatment. Prostate mpMRI is also currently used for detecting extracapsular extension (ECE) of PCa before surgery or radiotherapy, which is correlated with an increased risk of positive surgical margins and biochemical recurrence after treatment [40,41,42]. By applying machine-learning algorithms to detect ECE on prostate mpMRI images, Hou et al. [43] showed that it can improve the accuracy of ECE detection when compared with independent expert validation with an AUC of 0.72 (95% CI, 0.63–0.81, *p* < 0.05), a result that can be improved when adding entropy (a feature of textural analysis) data on an apparent diffusion coefficient (ADC) map, reaching AUC > 80% [44].

PSMA PET/CT has an established role in the evaluation of recurrent PCa and is increasingly incorporated into the primary staging of patients with newly diagnosed disease. Current guidelines recommend PSMA PET/CT, when available, for metastatic screening in high-risk localized and locally advanced PCa and support its use to improve staging accuracy in unfavorable intermediate-risk disease. PSMA PET/CT provides greater sensitivity for nodal and distant metastatic disease than conventional imaging, although limited spatial resolution means that microscopic lymph-node metastases may remain undetected. Its increasing use in primary staging therefore extends beyond the historically predominant application in biochemical recurrence [45]. PSMA PET/CT has historically been used extensively in biochemical recurrence, but its role has expanded substantially into primary staging, particularly in patients with unfavorable intermediate- and high-risk disease [45,46]. Beyond conventional visual interpretation, quantitative analysis of PSMA PET images may provide additional information regarding tumor phenotype and aggressiveness. Radiomics approaches have therefore been investigated in both primary and recurrent PCa. For example, Erle et al. [47], in a retrospective series of 72 patients, collected 77 radiomics features and, by using machine-learning classifiers to analyze the data, achieved the highest AUC of 0.95 with a sensitivity of 0.95 but a slightly lower specificity of 0.8 because of intense metabolic activity in other organs, which generated false positives in those regions, when compared with PET scans alone.

### 2.3. Integration of Radiomics in Clinical Pathway

The translation of radiomics and AI models from research into routine PCa care requires more than high discriminatory performance. Transparent reporting, appropriate validation, assessment of methodological quality and risk of bias, and evaluation of generalizability are essential before an AI model can be considered suitable for clinical implementation. TRIPOD+AI provides contemporary guidance for transparent reporting of clinical prediction models developed using regression or machine-learning methods, including description of the data source, study population, predictors and outcomes, model development, performance assessment, and validation [48]. PROBAST+AI complements reporting guidance by providing a structured framework for assessing methodological quality, risk of bias, and applicability of prediction models, including those developed using AI or machine-learning approaches [49]. Together with radiomics-specific frameworks such as RQS 2.0, these tools provide a more structured basis for interpreting the methodological robustness and potential clinical applicability of AI studies.

An important example of large-scale external benchmarking is the PI-CAI study by Saha et al. [50], an international paired non-inferiority confirmatory study evaluating AI for the detection of clinically significant PCa on MRI. The study included 10,207 MRI examinations from 9129 patients, with independent testing on 1000 examinations from multiple centers and a paired reader study involving 62 radiologists. In the reader study, the AI system achieved an AUROC of 0.91 compared with 0.86 for radiologists for detection of ISUP Grade Group ≥ 2 PCa. These findings provide substantially stronger evidence of generalizability than small single-center validation studies and demonstrate the value of benchmarking AI directly against clinical readers. Nevertheless, the study was based on retrospectively collected data, and prospective evaluation remains necessary to determine whether such performance translates into improved clinical decision-making and patient outcomes.

When considered against these principles, the evidence summarized in Table 1 remains heterogeneous. Several studies rely on retrospective single-center cohorts and internal validation or cross-validation, limiting assessment of model transportability. Studies incorporating independent external validation, including Hosseinzadeh et al. [51] and Bosma et al. [52], provide stronger evidence of generalizability, although external validation alone does not establish clinical utility. Large-scale benchmarking against clinical readers and existing standards of care therefore represents an important subsequent step in AI evaluation.

## 3. The Use of Pathomics in PCa Diagnosis and Treatment

### 3.1. What Is Pathomics?

Pathomics represents the evaluation and analysis of histopathological slides coming from prostate biopsies of whole-gland specimen extracted after prostatectomy that can be digitalized in order to extract quantitative data that describe PCa at a microscopic level [57]. Models such as glandular architecture, nucleolar structure and stromal component that are identified on histopathological slides can be correlated with tumor grade, aggressiveness and oncological patient outcomes [58]. The anatomical extent of PCa is described using the TNM classification, whereas clinical risk stratification integrates clinical stage, serum PSA level, and histopathological grade, currently reported using the ISUP Grade Group system. Each of these parameters provides complementary information but also has limitations: conventional imaging may fail to detect microscopic disease, histopathological grading is subject to interobserver variability, and PSA levels may be influenced by benign prostatic conditions and medical treatment. Pathomics may complement these established parameters by providing quantitative characterization of tumor morphology and facilitating integration with imaging and molecular biomarkers through AI and ML approaches [59,60].

### 3.2. Molecular Profiling, Imaging and Pathomics Integration for PCa Detection

An important potential advantage of digital pathology is its integration with molecular and genomic information to develop multimodal predictive models. Molecular alterations involving DNA damage repair genes, including BRCA1, BRCA2, and ATM, are associated with biologically aggressive subsets of PCa and may coexist with adverse histopathological features, including intraductal and cribriform morphology. Computational pathology may contribute to the identification and quantitative characterization of such morphological phenotypes and may ultimately assist in selecting cases for additional molecular testing. However, histomorphological or pathomic features should not currently be regarded as surrogate biomarkers of homologous recombination repair alterations or as independent predictors of response to PARP inhibitors; therapeutic eligibility and treatment selection rely on appropriate molecular/genomic testing [61]. Similarly, alterations involving androgen receptor signaling and ERG are important components of PCa biology and may be associated with particular molecular and morphological phenotypes. Although these relationships provide a rationale for integrating digital pathology with molecular profiling, the ability of ERG-associated morphological features identified by pathomics to predict response to androgen-directed therapy has not yet been established for clinical use [62].

In recent years, there has been a convergence of imaging studies (especially mpMRI and PET-CT) and pathomics for PCa detection. A suspected lesion identified on prostate mpMRI can be cross-checked with pathomics data, where digital pathology can identify nuclear atypia or architectural disorder, indicating a clinically significant PCa [63]. The integration of quantitative MRI and computational pathology represents a potential approach for improving risk stratification in patients undergoing active surveillance. However, the clinical utility of such multimodal AI models remains under investigation, and neither MRI progression nor AI-derived imaging or pathomic features should currently be regarded as independent indications for definitive treatment. MRI progression should prompt clinical reassessment and, when appropriate, histological reassessment in accordance with contemporary active-surveillance protocols. Prospective studies are required to determine whether AI-assisted approaches can eventually reduce unnecessary biopsies without compromising the detection of clinically significant disease [64]. A similar multimodal approach can integrate PSMA PET imaging with histopathological information. Associations have been reported between PSMA PET-derived imaging characteristics and adverse pathological features, including cribriform architecture and intraductal carcinoma [65]. Such findings are more appropriately considered a radiopathological correlation between molecular imaging and tissue morphology rather than a direct pathomic analysis of the PET signal. Further validation is required before these associations can be used for individual clinical decision-making [65].

Nagpal et al. [66] conducted a pioneer study in 2019 on a two-stage DLS based on a CNN for automated Gleason grading of prostatectomy specimens. The model was trained using 112 million pathologist-annotated image patches from 1226 slides and validated on an independent cohort of 331 patients. Compared with a reference standard established by genitourinary pathology experts, the DLS achieved significantly higher Grade Group classification accuracy than a cohort of 29 board-certified pathologists (70% vs. 61%, *p* = 0.002), with excellent discrimination for clinically relevant thresholds (AUC 0.95–0.96). Beyond Gleason grading, the algorithm provided more accurate quantification of Gleason patterns, generated fine-grained representations of tumor differentiation, and showed risk stratification performance approaching that of specialist pathologists, supporting the role of AI as a decision-support tool in digital pathology. Complementary evidence was subsequently provided by Pantanowitz et al., who evaluated a different clinical setting by developing and externally validating a deep-learning algorithm for the automated analysis of digitized prostate core biopsy specimens rather than radical prostatectomy specimens. Trained on more than 1.3 million annotated image patches, the model accurately detected PCa (external validation AUC = 0.991), differentiated low- from high-grade disease (AUC = 0.941), identified Gleason pattern 5 (AUC = 0.971), and detected perineural invasion (AUC = 0.957) that closely correlated with pathologist assessment. Importantly, the algorithm was successfully integrated into a routine pathology workflow as a second-reader quality assurance system, where it identified previously missed cancers while requiring minimal additional review time, highlighting the clinical feasibility of AI-assisted digital pathology [67]. Taken together, these studies demonstrate complementary applications of deep learning across the prostate pathology workflow: Nagpal et al. established its potential for automated Gleason grading and Grade Group classification in radical prostatectomy specimens, whereas Pantanowitz et al. demonstrated its utility for cancer detection, grading, tumor quantification, and identification of additional pathological features in prostate core biopsies.

Integration of AI and digitalization for PCa diagnosis is not devoid of limitations. First of all, differences in tissue fixation, processing, section thickness, and hematoxylin and eosin staining can alter image appearance, while variations in whole-slide scanners, magnification, illumination, color calibration, and image compression may introduce additional domain shifts between institutions. Tissue-related artifacts, including folds, tears, cautery effects, poor section quality, and out-of-focus regions, may further affect automated feature extraction and classification. Secondly, the quality of annotations used for model development represents another critical issue. AI algorithms generally learn from pathologist-generated labels, meaning that interobserver variability in Gleason pattern assessment and other morphological features may be propagated into the model itself. Expert consensus annotation and clearly defined histopathological reference standards are therefore important during model development. Furthermore, algorithms trained using material from a single institution may perform differently when applied to slides prepared and digitized in other laboratories. Robust external validation across institutions, scanners, staining protocols, and patient populations is therefore required before clinical implementation. In addition, PCa heterogeneity may affect AI training models, which will not be able to recognize rare features of PCa. Moreover, integration of AI in clinical practice is subject to technical challenges, such as resolution differences between MRI machines, and aggressive tumor features that are difficult to interpret may cause gaps in the AI diagnostic precision [68]. Lastly, ethical aspects that focus on patient consent, data privacy, equity, transparence and the impact on pathology or radiology specialists should be taken into consideration before the adoption of AI and digital pathology [69,70,71].

## 4. Conclusions

Our narrative review illustrates that AI is rapidly reshaping the diagnostic and therapeutic landscape of prostate cancer by enabling the extraction of clinically relevant information from radiological and histopathological data that extends beyond conventional human interpretation. Radiomics has demonstrated considerable potential for improving prostate cancer detection, lesion characterization, risk stratification, prediction of extracapsular extension, and treatment planning across multiple imaging modalities. Although multiparametric MRI currently provides the most robust platform for radiomics applications, ultrasound, CT, and PSMA PET radiomics have also shown encouraging results in selected clinical settings. Similarly, pathomics has shown promising performance for automated tumor detection, grading, quantification, and identification of adverse pathological features. In selected retrospective validation and reader studies, AI-based systems have achieved diagnostic performance comparable to, and in some settings exceeding, that of participating pathologists. However, these findings are study-specific and should not be interpreted as evidence of equivalent or superior performance in routine clinical practice.

However, high diagnostic accuracy or discrimination reported in retrospective studies should not be equated with demonstrated clinical benefit. Improvements in AUC, sensitivity, or specificity do not necessarily translate into fewer unnecessary biopsies, more appropriate treatment selection, improved quality of life, or better oncological outcomes. Demonstrating such benefits will require prospective evaluation of AI-assisted strategies against current standards of care using clinically meaningful patient-level endpoints.

Despite these advances, the transition of AI from research to routine clinical practice remains challenging. Most currently available models have been developed using retrospective, single-center datasets with heterogeneous imaging protocols, manual segmentation, and limited external validation. Standardization of image acquisition, feature extraction, annotation strategies, and reporting methodology, together with adherence to quality assessment frameworks such as the Radiomics Quality Score (RQS 2.0), will be essential to ensure reproducibility and facilitate clinical adoption. In parallel, ethical, regulatory, and medicolegal considerations, including data privacy, algorithm transparency, explainability, and responsibility for AI-assisted clinical decisions, must be carefully addressed.

The future development of AI in PCa is likely to involve multimodal models integrating radiomics, pathomics, molecular and genomic biomarkers, and clinical variables into unified decision-support systems. Such approaches have the potential to improve the detection and characterization of clinically significant disease and refine risk stratification and active-surveillance assessment. However, these potential benefits remain to be demonstrated in prospective multicenter studies and, ultimately, in real-world clinical practice using endpoints that extend beyond algorithmic performance to include effects on clinical decision-making, patient outcomes, resource utilization, and quality of life.

At present, AI should therefore be regarded as a promising complementary decision-support technology rather than a replacement for clinical expertise. Its clinical value will depend not only on diagnostic accuracy and reproducibility but also on external generalizability, integration into existing workflows, interpretability, cost-effectiveness, and evidence that AI-assisted care provides meaningful benefit to patients with PCa.

## Figures and Tables

**Table 1:** Overview of representative radiomics and artificial intelligence studies for prostate cancer detection, characterization, and prognostic assessment.

Study (Year)	Study Design/Cohort	Imaging Modality	Method/ AI Approach	Clinical Endpoint/ Reference Standard	Validation and Performance	Key Results
Xiong et al. (2021) [44]	Retrospective, Single-center study/85 patients	bpMRI: T2WI + DWI/ADC	Hand-crafted MRI texture/radiomic features; first-order and GLCM features; multivariable logistic regression	Prediction of high-grade PCa, defined as GS ≥ 7 vs. GS < 7; TRUS-guided biopsy histopathology	Internal analysis; no independent external validation. Best individual feature: ADC entropy, AUC 0.800; combined ADC kurtosis + skewness + entropy, AUC 0.846.	ADC-derived radiomic features significantly outperformed T2WI features for predicting high-grade prostate cancer.
Osman et al. (2019) [33]	Retrospective, single-center study/342 patients	Non-contrast radiotherapy planning CT	CT radiomics; feature filtering followed by LASSO/Elastic Net regularization	Gleason grade and clinical risk stratification; biopsy-derived Gleason score and NICE risk classification	Internal train/test assessment with repeated cross-validation; no external cohort. Training AUCs up to 0.90–1.00 for several classification tasks; validation performance lower, including 0.75 for low- vs. high-risk and 0.70 for GS 7 vs. >7	This was the first study to investigate radiomics derived from routine non-contrast planning CT for prostate cancer risk stratification. CT radiomics accurately differentiated low- from high-grade disease and low- from high-risk patients.
Bosetti et al. (2020) [34]	Retrospective, single-center study/31 patients	Longitudinal radiotherapy CBCT	Hand-crafted longitudinal CBCT radiomics; logistic regression	Prediction of tumor stage, GS, PSA category, NCCN risk group and biochemical recurrence; clinical/pathological variables and follow-up	Repeated 3-fold cross-validation; no independent test cohort. Risk group AUC 0.83; T stage 0.78–0.80; GS classification 0.80–0.82; PSA <10 ng/mL 0.8	Radiotherapy energy- and shape-derived features were the strongest predictors of tumor aggressiveness.
Giannini et al. (2021) [53]	Retrospective, single-center study/90 patients	mpMRI	SVM-based CAD, generating voxel-wise probability maps; not a conventional radiomics prediction model	Detection of PCa/csPCa; pathology used for cancer cases, with clinical follow-up supporting negative cases	Multi-observer crossover evaluation; no external multicenter validation. CAD-assisted reader AUCs 0.778–0.889 vs. 0.796–0.871 without CAD	CAD improved per-patient sensitivity for clinically significant prostate cancer (Gleason score > 6) from 68.7% to 78.1% (*p* = 0.018) without a statistically significant reduction in specificity (94.8% vs. 89.6%, *p* = 0.072).
Hosseinzadeh et al. (2022) [51]	Retrospective, multicenter study/2734 patients	bpMRI: T2WI + ADC + high-b-value DWI	Two-stage deep-learning CAD using U-Net-based zonal segmentation and lesion detection	Detection of csPCa, GG ≥ 2 (GS ≥ 3 + 4); external reference standard based on systematic and MRI-targeted biopsy	Internal test cohort plus independent external-center validation (n = 296) with histopathological reference standard. External AUC 0.85 (reported in manuscript as 0.849); sensitivity 85% at 1 FP/patient	The study demonstrated that AI performance remained strongly dependent on training data size, with performance continuing to improve even after nearly 2000 training examinations.
Hectors et al. (2021) [54]	Retrospective, single-center/240 patients with PIRADS 3 lesions	T2-weighted MRI. Each PI-RADS 3 index lesion underwent manual three-dimensional segmentation	Hand-crafted T2WI radiomics + Random Forest machine learning	Prediction of csPCa in PI-RADS 3 lesions, defined as Grade Group ≥2 on targeted biopsy or corresponding systematic biopsy	Independent chronological internal test cohort; no external validation. Test AUC 0.76; sensitivity 75.0%, specificity 79.6%; PSA density AUC 0.61.	The radiomics model was the only statistically significant predictor of csPCa among the evaluated variables, and adding PSA density or prostate volume did not improve diagnostic performance.
Li et al. (2024) [55]	Retrospective, single-center/231 patients	bpMRI	Deep transfer learning, ResNet50; comparison of 2D and 2.5D approaches	Prediction of csPCa/aggressiveness; histopathology, with csPCa defined as GS ≥ 7 and non-csPCa as GS 3 + 3	Random internal train/test split; no external validation. Combined 2.5D T2WI+ADC: training AUC 0.960, test AUC 0.949	The study demonstrated that incorporating adjacent MRI slices (2.5D segmentation) significantly improved automated prediction of prostate cancer aggressiveness compared with conventional single-slice (2D) deep learning.
Papp et al. (2021) [56]	Prospective, single-center/52 patients	[68Ga]Ga-PSMA-11 PET/MRI, ADC and T2WI	Radiomics + ensemble supervised machine learning, including Random Forest classifiers	Low- vs. high-risk lesion classification, biochemical recurrence and overall patient risk; radical-prostatectomy pathology and clinical follow-up	1000-fold Monte Carlo cross-validation; no external validation. Lesion risk model AUC 0.86; BCR model 0.90; overall patient risk model 0.94	The study demonstrated that PSMA PET/MRI radiomics combined with supervised machine learning enables accurate non-invasive characterization of clinically significant prostate cancer lesions and prediction of biochemical recurrence and overall patient risk without relying solely on biopsy-derived Gleason grading.
Bosma et al. (2023) [52]	Retrospective, multicenter study/7756 biparametric prostate MRI examinations from 6380 patients.	bpMRI	Report-guided semi-supervised deep learning (RG-SSL) using nnU-Net and NLP-derived report information to generate pseudo-labels	Detection of csPCa; training guided by PI-RADS ≥ 4 findings, while the external test reference standard was histopathologically confirmed GGG ≥ 2 by biopsy and/or prostatectomy.	Five-fold CV during development plus independent external-center validation. With 100/300/1000/3050 manual labels, RG-SSL AUC 0.86/0.88/0.89/0.89, vs. supervised learning 0.78/0.79/0.84/0.87	The study demonstrated that diagnostic radiology reports can be successfully leveraged to generate high-quality pseudo-labels, substantially reducing the need for costly voxel-level manual annotations while maintaining expert-level diagnostic performance.

## Data Availability

No new data were created or analyzed in this study. Data sharing is not applicable to this article.
